# Reducing Suppressors of Cytokine Signaling-3 (SOCS3) Expression Promotes M2 Macrophage Polarization and Functional Recovery After Intracerebral Hemorrhage

**DOI:** 10.3389/fneur.2020.586905

**Published:** 2020-11-12

**Authors:** Xin-Chao Ji, Ya-Jun Shi, Yan Zhang, Ming-Ze Chang, Gang Zhao

**Affiliations:** ^1^Department of Neurology, Xi'an No. 3 Hospital, The Affiliated Hospital of Northwest University, Xi'an, China; ^2^Department of Neurology, Xijing Hospital, Fourth Military Medical University, Xi'an, China; ^3^Affiliated Bayi Brain Hospital, The Seventh Medical Center of PLA General Hospital, Beijing, China

**Keywords:** intracerebral, hemorrhage, SOCS3, microglia/macrophage polarization, nuclear factor-κB, functional recovery

## Abstract

Intracerebral hemorrhage (ICH) is a fatal subtype of stroke, and effective interventions to improve the functional outcomes are still lacking. Suppressor of cytokine signaling 3 (SOCS3) plays critical roles in the inflammatory response by negatively regulating cytokine-Jak–Stat signaling. However, the role of SOCS3 in the regulation of macrophage polarization is highly controversial and the fine regulation exerted by SOCS3 needs further understanding. In this study, rat ICH models were established by infusion of collagenase into the caudate nucleus. To decrease SOCS3 expression into microglia/macrophages in the hemorrhagic lesion area, we injected lentiviral short hairpin RNA (shSOCS3) (Lenti-shSOCS3) into the hematoma cavity at 24 h following ICH. We found that the number of iNOS-positive cells (M1 phenotype) was significantly reduced, whereas arginase-1-positive cells (M2 phenotype) were markedly elevated in animals that received Lenti-shSOCS3 injections compared with those in the Lenti-EGFP and saline groups. The increase in arginase-1-positive cells was associated with a significantly lower pro-inflammatory microenvironment, which included the downregulation of pro-inflammatory cytokines [interleukin (IL)-1β, IL-6, and TNF-α] and concurrent upregulation of anti-inflammatory (IL-10) mediators. In addition, this marked shift toward the M2 phenotype was associated with suppressed NF-κB activation. Furthermore, these changes notably enhanced the neuroprotective effects and functional recovery in Lenti-shSOCS3-injected animals. Our findings indicated that reduction in SOCS3 expression caused a marked bias toward the M2 phenotype and ameliorated the inflammatory microenvironment, which enhanced neuroprotective effects and resulted in notable improvement in functional recovery after ICH.

## Introduction

Microglia and hematogenous macrophages are central modulators in the local inflammatory response after CNS injury (1). In response to different environmental stimuli, microglia/macrophages have distinct subtypes (2): “classically activated” macrophages (M1) induced by lipopolysaccharide (LPS) or interferon-gamma (IFNγ), which represent a pro-inflammatory phenotype that produces high levels of pro-inflammatory mediators such as nitric oxide, superoxide, tumor necrosis factor alpha (TNF-α), interleukin 12, and 23 (IL-12, IL-23) (3). These cells exhibit enhanced antimicrobial and cytotoxic properties. Conversely, IL-4 and IL-13 promote the differentiation of “alternatively activated” macrophages (M2). These cells are characterized as anti-inflammatory phenotype and promote tissue remodeling (4). In addition, it has been suggested that M1 polarization is toxic to neurons, while M2 cells facilitate tissue repair (5). To date, most studies on microglial/macrophage phenotypes have focused on traumatic brain injuries, cerebral ischemia, and spinal cord injury (6–10). However, the polarization of microglia/macrophages following intracerebral hemorrhage (ICH) has not been well-studied.

Suppressor of cytokine signaling (SOCS) proteins play critical roles in the inflammatory response by negatively regulating cytokine-JAK–STAT signaling (11, 12). It has been suggested that SOCS3, one of the most studied members of the SOCS family, is an important regulator of macrophage function. As described, SOCS3-deficient macrophages exhibit marked anti-inflammatory properties and myeloid-restricted SOCS3 knockout mice showed enhanced resistance to LPS-induced endotoxic shock (13). Paradoxically, Qin et al., reported that SOCS3-deleted macrophages had higher levels of the M1 genes IL-1b, IL-6, IL-12, IL-23, and iNOS and exacerbated LPS-induced sepsis in LysMCre-SOCS3^*fl*/*fl*^ mice which lacked SOCS3 in myeloid lineage cells (14). These conflicting findings have led to confusion regarding the role of SOCS3 in macrophage phenotype differentiation. To further investigate the effect of SOCS3 in the regulation of microglial/macrophage polarization and the inflammatory response, we utilized lentiviral delivery of short hairpin RNA (shSOCS3) (Lenti-shSOCS3) into the hematoma cavity to knock down SOCS3 expression following ICH. Our study showed that SOCS3-silenced microglia/macrophages have a marked bias toward the M2 phenotype and obvious anti-inflammatory traits, which led to a notable functional recovery after ICH.

## Materials and Methods

### Animals

Healthy, adult, male, Sprague Dawley (SD) rats, weighing 250–300 g, which were obtained from the Experimental Animal Center of the Fourth Military Medical University were used in this study. All rats were housed under standard laboratory conditions on a 12-h light–dark cycle and allowed free access to food and water. All experiments were reviewed and approved by the Institutional Animal Care and Use Committee of the Fourth Military Medical University PLA. The rats were randomly assigned to four groups: sham, saline control (ICH with injection saline), lentiviral vector control (ICH with injection Lenti-EGFP), and Lenti-shSOCS3 (ICH with injection Lenti-shSOCS3).

### ICH Models

ICH was induced by collagenase as previously described (15). SD rats were anesthetized with 3.6% chloral hydrate and placed in a stereotaxic device. Fur was shaved and the scalp was incised, after which sphenotresia was performed with a dental drill. Collagenase VII (0.4 U in 0.8 μL normal saline, Sigma-Aldrich) was injected into the right caudate nucleus (coordinates from bregma: AP 0.5 mm, ML 3.0 mm, DV 5.5 mm). Collagenase was continuously injected for 4 min at a speed of 200 nL/min with a 5-μL Hamilton syringe. The needle was then held in place for another 10 min to prevent backflow. After the injections were completed, the skin overlying the skull was sutured with 4–0 silk suture. Sham-operated animals underwent the same surgical procedures without the collagenase injection.

### Vector Injections

LVs U6-MCS-Ubi-EGFP SOCS3 small hairpin RNA (Lenti-shSOCS3), which target the sequence TGCAGGAGAGCGGATTCTA (NM 053565.1), were generated. Twenty four hours post-ICH, 4 μL of viral solution was stereotaxically delivered into the hematoma cavity at the same site of collagenase injection, as stated above (coordinates from bregma: AP 0.5 mm, ML 3.0 mm, DV 5.5 mm), at a rate of 200 nL/min with a 5-μL Hamilton syringe. The needle was left in place for an additional 5 min and then gently withdrawn. The same dose of lentiviral vector which expresses EGFP alone (Lenti-EGFP) was also injected into the hematoma cavity as the lentiviral vector control. For saline control, the same dose of normal saline was injected into the hematoma cavity.

### Behavioral Assessments

Neurological abnormalities were assessed according to a modified neurological severity score (mNSS). The animals in each group (*n* = 10) were tested at 1, 3, 7, and 14 days post-ICH by two independent investigators blinded to the experimental condition.

### Preparation for Histology and Immunohistochemistry

Animals were anesthetized with 3.6% chloral hydrate and transcardially perfused with ice-cold phosphate-buffered saline (PBS) followed by 4% paraformaldehyde (PFA) in 0.1 M PBS. Sagittal sections (14 μm) were collected with a Leica freezing microtome. Brain sections were blocked (10% bovine serum albumin and 0.3% Triton X-100 in PBS) for 1 h at room temperature and then incubated with primary antibodies: anti-Iba1 (1:500; Wako, 019-19741), anti-arginase-1 (1:100; Abcam, ab60176), anti-iNOS (6 μg/mL; R&D Systems, MAB9502), anti-GFAP (1:2,000; Abcam, ab7260), anti-NeuN (1:200; Abcam, ab177487), and anti-NF-200 (2 μg/mL; Abcam, ab82259) overnight at 4°C. The sections were washed three times and then incubated with Alexa Fluor secondary antibody (1:1,000; Invitrogen) for 1 h at room temperature. Counter staining was performed with 40,6-diamidino-2-phenylindole, and images were captured by confocal microscopy (Leica, SP5II). To quantify the immune-positive cells and area, five representative sagittal sections were serially selected (1:6) around the center of the hematoma cavity (*n* = 4 for each group). Four images were captured in each section at 200× magnification with confocal microscopy. The immuno-positive cells and area were quantified with NIH ImageJ software.

### Enzyme-Linked Immunosorbent Assay (ELISA)

At 7 and 14 days post-ICH, the animals (*n* = 4 per group) were sacrificed and the peri-hematoma brain tissue was obtained. The samples were homogenized immediately and then centrifuged at 5,000 × g for 15 min at 4°C to remove cellular debris. The concentrations of TNF-α, IL-1β, IL-6, and IL10 were detected with ELISA kits according to the manufacturers' instructions (Invitrogen). The results were expressed as pg/mg.

### Western Blotting

Lysates from brain tissues of the peri-hematomal region were subjected to SDS-PAGE and then were electrophoretically transferred onto a polyvinylidene fluoride membrane (Merck Millipore). After blocking with 5% non-fat milk, the membranes were incubated overnight at 4°C with primary antibodies as follows: SOCS3 (Abcam), phosphorylated Stat3 (Tyr705), Stat3, NF-κB p65, p-NF-κB, and p65 (Cell Signaling Technology). Membranes were then washed with Tris-buffered saline containing Tween 20 and incubated with HRP-conjugated secondary antibodies (Sigma-Aldrich) for 2 h at room temperature. An ECL detection kit (Thermo Fisher Scientific) was used to visualize the immune-reactive proteins. The intensities of the bands were analyzed with NIH ImageJ software.

### Statistical Analysis

Comparisons between two groups were made by independent samples *t*-test. Statistical significance between multiple groups was determined using ANOVA followed by Tukey's test. For behavioral testing, data were analyzed using two-way repeated-measures ANOVA followed by Bonferroni's post-test. Statistical data are presented as mean ± SD and performed using SPSS 20.0. Significance was accepted at *P* < 0.05.

## Results

### Confirmation of Lenti-shSOCS3 Expression

**Figure 1:**
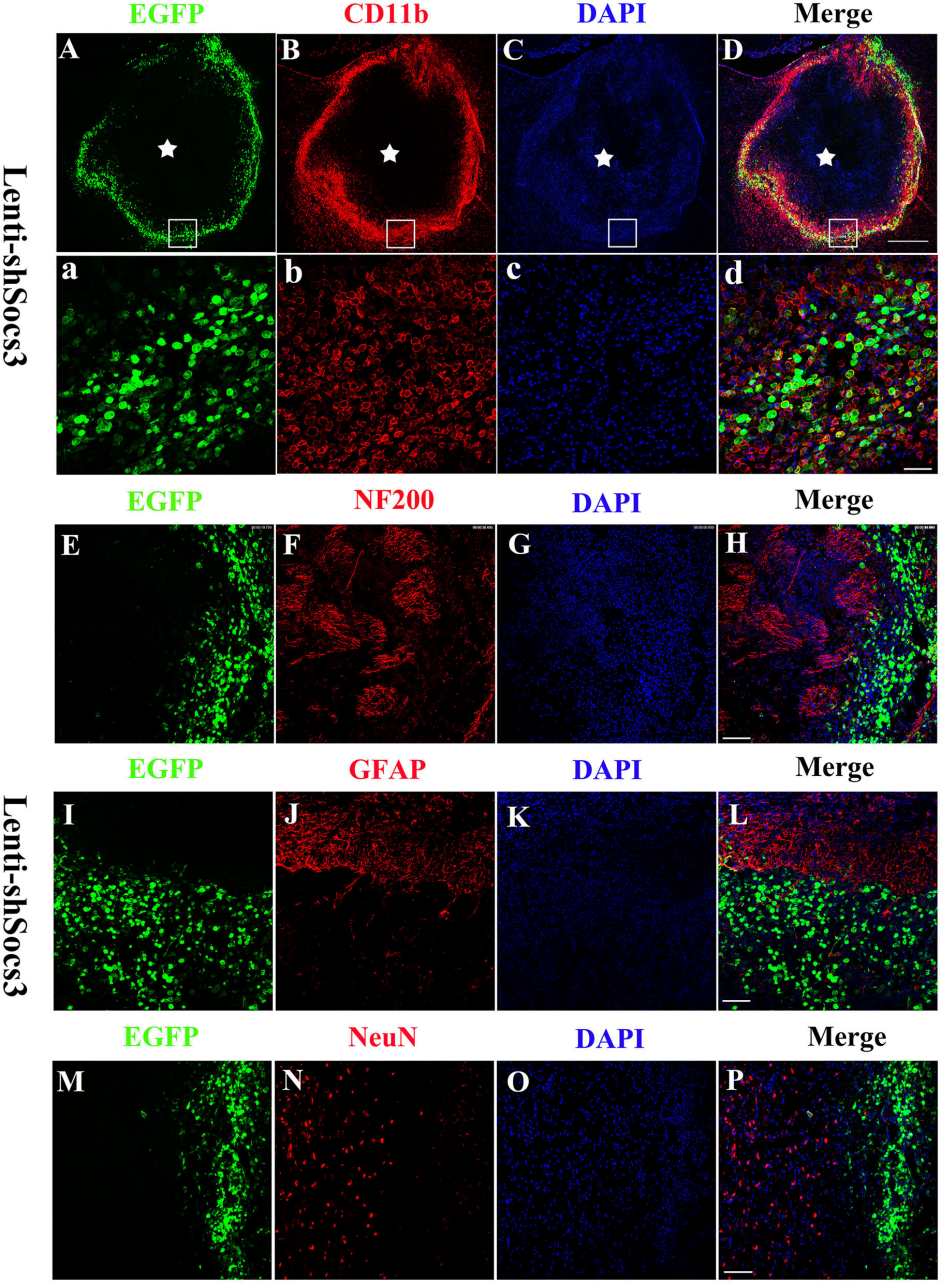
Lenti-shSOCS3-infected microglia/macrophages in the hemorrhagic lesion area. Confocal microscopy revealed that EGFP-positive cells were distributed around the edge of the hematoma cavity at 7 days post-ICH. [**(A–D)** Scale bar = 500 μm, pentagram indicates the epicenter of the hematoma cavity]. The infected EGFP-positive cells stained positively for CD11b [a microglial/macrophage marker, **(a–d)** scale bar = 50 μm], but not NF200 [a neurofilament marker; **(E–H)** scale bar = 100 μm], GFAP [an astrocytic marker; **(I–L)** scale bar = 100 μm], and NeuN-positive cells [a neuronal marker; **(M–P)** scale bar = 100 μm].

Seven days after ICH, confocal microscopy revealed that EGFP-positive cells were distributed around the edge of the hematoma cavity (Figures 1A–D). To confirm the identity of the GFP-positive cells, sections were labeled with CD11b (a macrophage/microglia marker Figures 1a–d), NF200 (a neurofilament marker; Figures 1E–H), GFAP (an astrocytic marker; Figures 1I–L), and NeuN (a neuronal marker; Figures 1M–P) by immunofluorescent staining. The data showed that the majority of the EGFP-positive cells stained positively for CD11b, but not for NF200, GFAP, and NeuN in the Lenti-EGFP and Lenti-BDNF groups. These results indicate that Lenti-shSOCS3 mainly infected microglia/macrophages in the hemorrhagic lesion area.

### Reduction of SOCS3 Expression Promotes Microglia/Macrophage Shift From M1 to M2 Polarization

**Figure 2:**
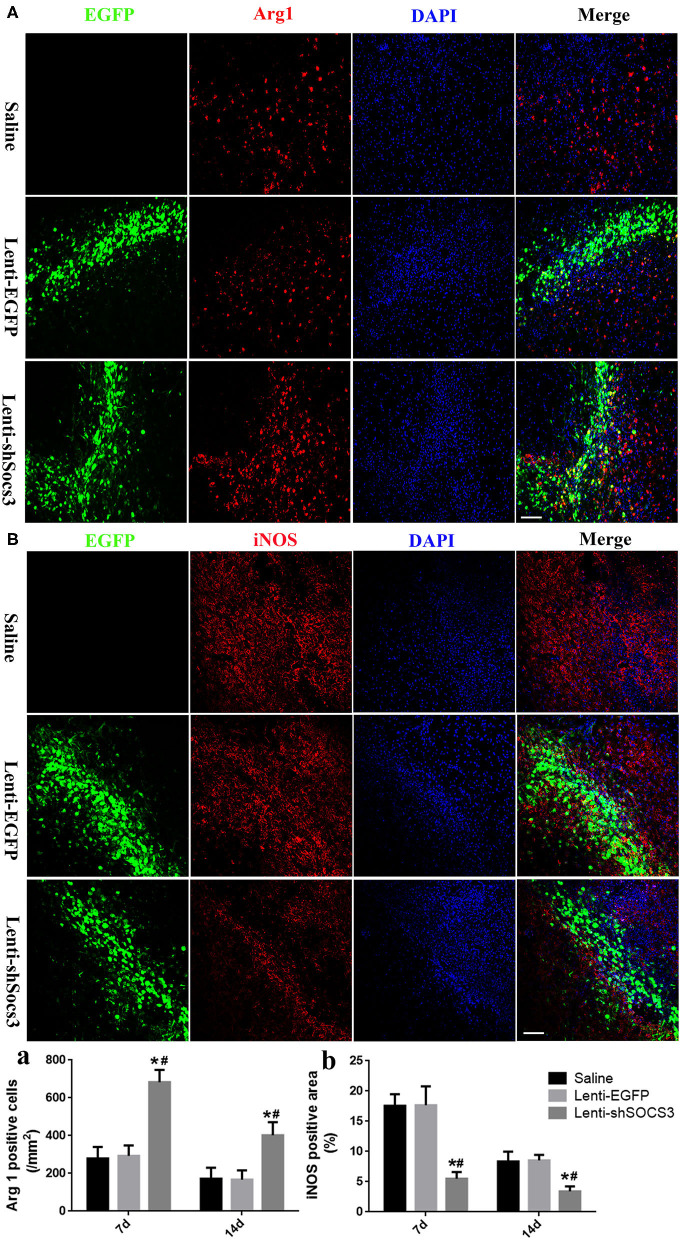
Reduction of SOCS3 expression promotes the microglial/macrophage polarization from the M1 to M2 phenotype. The expression profiles of microglial/macrophage phenotypes were evaluated by immunofluorescence staining with Arg1 **(A)** and iNOS **(B)** at 7 and 14 days post-ICH. Quantitative analysis of the number of Arg1-positive cells **(a)** and the iNOS-positive areas **(b)** in each group. Scale bar = 100 μm. Data are presented as mean ± SD (*n* = 4 mice per group). *P < 0.05 vs. saline group, ^#^*P* < 0.05 vs. Lenti-EGFP group.

To investigate the effects of SOCS3 silencing on microglial/macrophage polarization, the expression profiles of the microglia/macrophage phenotype were evaluated by immunofluorescence staining with iNOS and arginase-1 associated with the M1 and M2 phenotypes, respectively, at 7 and 14 days post-ICH. The results showed that higher numbers of arginase-1-positive cells were found in the Lenti-shSOCS3 group compared with the Lenti-EGFP and saline groups (Figures 2A,a). In addition, the iNOS-positive area was significantly reduced in mice that received Lenti-shSOCS3 injections compared with mice in Lenti-EGFP and saline groups both (Figures 2B,b).

### Effects of Lenti-shSOCS3 on Cytokine Expression

**Figure 3:**
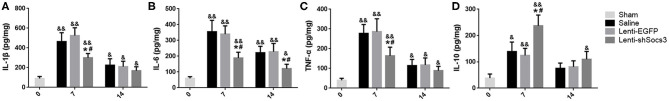
Reduced SOCS3 expression ameliorates the inflammatory microenvironment. The levels of inflammatory cytokines were detected by ELISA. The production of IL-1β **(A)**, IL-6 **(B)**, and TNF-α **(C)** were significantly reduced in the Lenti-shSOCS3 group at 7 and 14 days post-ICH. In contrast, the expression of IL-10 **(D)** was significantly elevated in the Lenti-shSOCS3 group compared with the Lenti-EGFP and saline groups at 7 days post-ICH. Data are presented as mean ± SD (*n* = 4 mice per group). ^&^*P* < 0.05 (^&&^*P* < 0.01) vs. sham group, *P < 0.05 vs. saline group, ^#^*P* < 0.05 vs. Lenti-EGFP group.

It has been suggested that pro-inflammatory cytokines (IL-1β, IL-6, and TNF-α) are produced specifically by M1 macrophages, while the anti-inflammatory cytokine, IL-10, is associated with M2 macrophages. To evaluate the effects of Lenti-shSOCS3 infection on the inflammatory cytokine profile after ICH, the levels of inflammatory cytokines were detected by ELISA. The data showed that the production of IL-1β, TNF-α, and IL-6 were significantly reduced in mice that received Lenti-shSOCS3 injections compared with mice in the Lenti-EGFP and saline groups at 7 and 14 days post-ICH (Figures 3A–C). In contrast, the expression of IL-10 was significantly elevated in the Lenti-shSOCS3 group compared with the Lenti-EGFP and saline groups at 7 days post-ICH (Figure 3D). These data suggest that reduction of SOCS3 expression in microglia/macrophages ameliorates the inflammatory microenvironment after ICH.

### Decreased SOCS3 Expression Suppresses the Activation of NF-κB

**Figure 4:**
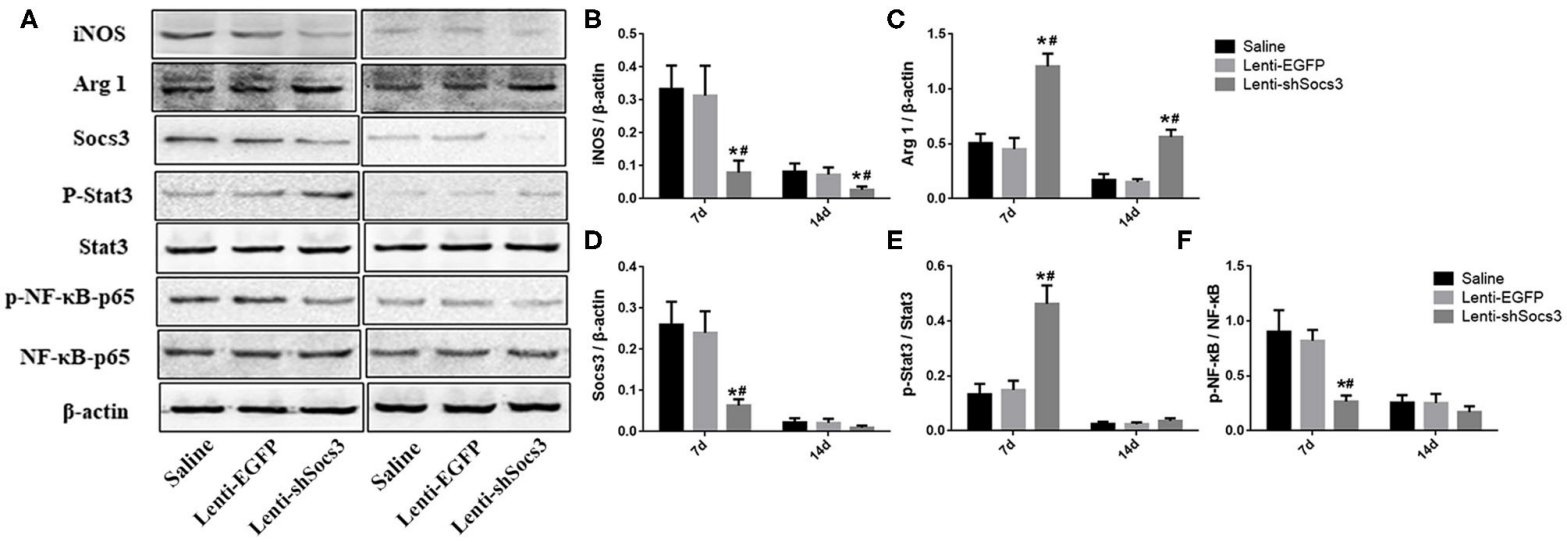
Decreased SOCS3 expression suppresses the activation of NF-κB. Western blot analyses of SOCS3, p-Stat3/Stat3, and p-NF-κB p65/NF-κB p65 were performed at 7 and 14 days post-ICH **(A)**. Densitometric analysis of iNOS **(B)**, Arg1 **(C)**, SOCS3 **(D)**, p-Stat3/Stat3 **(E)**, and p-NF-κB p65/NF-κB p65 **(F)**. Data are presented as mean ± SD (*n* = 4 mice per group). *P < 0.05 vs. saline group, ^#^*P* < 0.05, vs. Lenti-EGFP group.

To further confirm macrophage/microglial polarization around the hematoma cavity after ICH, the protein expression levels of iNOS and Arg1 were quantified by using western blot (Figure 4A). In line with the immunofluorescence results shown in Figure 2, the level of Arg1 was significantly increased and iNOS was markedly reduced in the Lenti-shSOCS3 group compared with the Lenti-EGFP and saline groups at 7 and 14 days post-ICH (Figures 4B,C).

As documented, SOCS3 is the negative feedback molecule of Stat3. Furthermore, NF-κB is a key transcription factor related to M1 microglial/macrophage activation, which regulates the expression of most M1-associated genes (IL-1β, IL-6, TNF-α, cyclooxygenase 2, and IL12p40). To further illustrate the underlying mechanism in the alteration of macrophage polarization, the activation of the SOCS3/Stat3 and NF-κB pathways were detected (Figure 4A). The results showed that the level of SOCS3 was significantly decreased in mice in the Lenti-shSOCS3 groups compared with mice in the Lenti-EGFP and saline groups (Figure 4D), while the level of phosphorylation of Stat3 was significantly increased in the Lenti-shSOCS3 group at 7 days post-ICH (Figure 4E). In addition, the level of phosphorylated NF-κB p65 was significantly reduced in the Lenti-shSOCS3 group compared with the Lenti-EGFP and saline groups at 7 days post-ICH (Figure 4F). These results indicate that knockdown SOCS3 expression in microglia/macrophages inhibited the activation of the NF-κB pathway.

### Reduction of SOCS3 Expression Enhances Neuroprotective Effects and Functional Recovery

**Figure 5:**
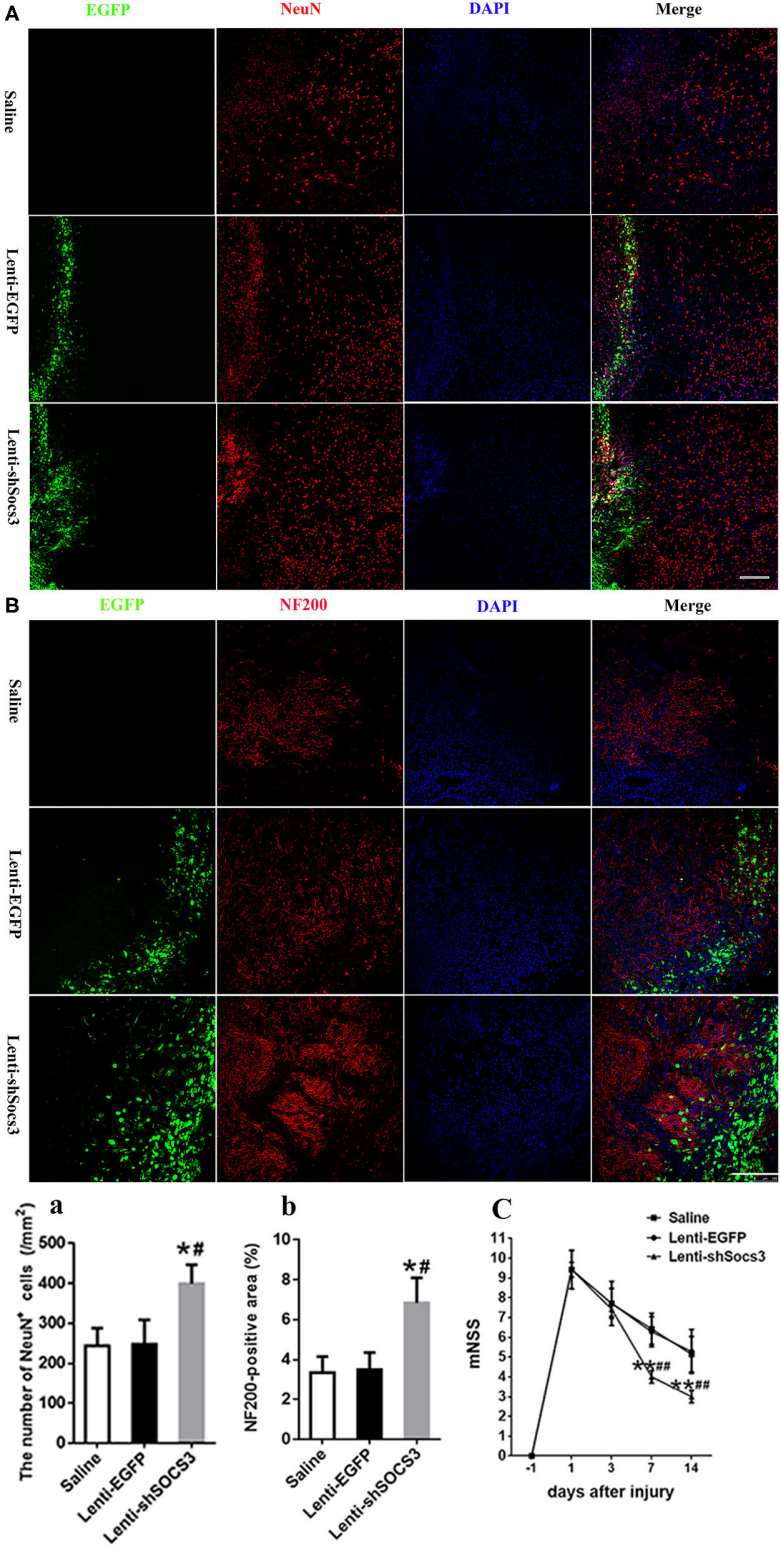
Reduction of SOCS3 expression promotes neuronal survival and functional recovery. **(A, a)** Quantitative analysis of the number of NeuN-positive cells around the hematoma at 14 days post-ICH (scale bar = 200 μm; *n* = 4 per group). (**B, b)** Quantitative analysis of the NF200-positive areas at the lesion epicenter, at 14 days after ICH (scale bar = 200 μm; *n* = 4 per group). **(C)** Time course of functional recovery was examined with modified neurological severity scores (mNSS) at 1, 3, 7, and 14 days post-ICH (*n* = 10 per group). All measures are presented as mean ± SD. *P < 0.05 vs. saline group, ^#^*P* < 0.05 vs. Lenti-EGFP group.

The data showed that a significantly greater number of NeuN-positive neurons were observed in the Lenti-shSOCS3 group compared with the Lenti-EGFP and saline groups at 7 and 14 days post-ICH (Figures 5A,a). In addition, a greater abundance of NF200-positive nerve fibers was seen in the Lenti-shSOCS3 group compared with the Lenti-EGFP and saline groups (Figures 5B,b). Furthermore, the time course of functional recovery and the score of each animal at 4 weeks post-ICH were evaluated using the mNSS. Significant functional recovery was observed in mice that received Lenti-shSOCS3 injections compared with mice in the Lenti-EGFP and saline groups at 7–28 days post-ICH (Figure 5C). These results indicate that a marked microglial/macrophage transition to the M2 phenotype enhanced neuroprotective effects and contributed to the locomotor functional recovery post-ICH.

## Discussion

ICH is a common acute cerebrovascular disease with high mortality and disability rates throughout the world. Although dramatic progress has been made to decrease mortality rates, effective treatment to significantly improve ICH prognosis is still unavailable (16–18). During the initial phases of ICH, a rapid accumulation of blood within the brain parenchyma leads to herniation and cell death, referred to as primary brain damage. Subsequently, the products of erythrocyte lysis trigger complex secondary brain injury (SBI) including local inflammatory responses, reactive gliosis, neuronal apoptosis, and necrotic death (19, 20). It has been demonstrated that inflammation plays a pivotal role in the pathophysiology of ICH-induced SBI and therefore has been regarded as a “double-edged sword” for neurological recovery. On the one hand, the initial response promotes tissue repair by clearing erythrocyte lysis. On the other hand, these beneficial effects may be overshadowed by the excessive accumulation of pro-inflammatory cytokines and neurotoxins. Accumulating studies suggest that microglial/macrophage polarization might contribute to the conflicting effects of the inflammatory response in ICH.

At the lesion site of IHC, activated microglia and hematogenous macrophages are key players in the inflammatory response during SBI. It has been well-documented that microglia and macrophages are characterized by marked heterogeneity and are generally classified into two subsets: pro-inflammatory phenotype (M1) and alternatively activated macrophages (M2). Furthermore, M2 phenotype macrophages are transient during early injury and are markedly reduced by 7–14 days after ICH. However, M1 phenotypic macrophages are upregulated later than the M2 phenotype. They are longer lasting and eventually predominate at the lesion site (21). Here, we demonstrated that Lenti-SOCS3-infected microglia/macrophages exhibited a marked bias toward the M2 phenotype at 7 and 14 days post-ICH, which ameliorated the inflammatory microenvironment. There was a marked downregulation of pro-inflammatory cytokines and a corresponding upregulation of anti-inflammatory mediators after IHC. These changes were also associated with notable neuroprotective and functional recovery in Lenti-SOCS3-injected mice. The results of the present study indicated that SOCS3 is an important regulator of microglial/macrophage polarization.

SOCS3 hampers the activation of Stat3-mediated signaling by regulating different cytokines and growth factors and by having distinct roles in the regulation of the inflammatory response depending on its expression profile in various cell types (22, 23). IL-6, as a pro-inflammatory cytokine, has been proposed to contribute to the progression of rheumatoid arthritis and Crohn's disease, while SOCS3 is a major negative regulator of the IL-6-related cytokine-STAT3 pathway and enhanced expression of SOCS3 inhibits these inflammatory responses (11, 24, 25). These data indicate that SOCS3 likely plays an anti-inflammatory role in non-immune cells. However, on the contrary, Yasukawa et al. demonstrated that IL-6 induced an IL-10-like anti-inflammatory response in the SOCS3-deficient macrophage and that SOCS3^*fl*/*fl*^ LysMcre mice were protected from the lethal effects of LPS (26). Furthermore, SOCS3-deficient macrophages and dendritic cells infected with M. tuberculosis or BCG show decreased TNF-α and IL-12 production (27). It was explained that although both IL-6 and IL-10 activate Stat3 after binding to their respective receptors, prolonged activation of Stat3 by IL-10 is required to suppress LPS signaling; the response to IL-6 is only transient due to the induction of SOCS3. However, based on our findings, we believe that the anti-inflammatory effects of silencing SOCS3 were due to the changes in macrophage polarization toward the M2 phenotype.

Furthermore, as a key transcription factor, activated NF-κB induces the transcription of many pro-inflammatory genes (COX-2, TNF-α, IL-1β, IL-6, iNOS, and IL-12) and plays a key role in M1 polarization (28–30). The activity of NF-κB is rapidly induced in response to pro-inflammatory stimuli and results in the proteasomal degradation of IκBs and the release of NF-κB p65/p50 heterodimers from the NF-κB/IκBs complex. Activated NF-κB then translocates into the nucleus and induces the transcription of many pro-inflammatory genes. Our data indicate that the level of phosphorylation of NF-κB p65 was significantly decreased in Lenti-shSOCS3-injected mice post-ICH. These results indicate that the shift toward the M2 phenotype was due, at least in part, to the inhibition of the NF-κB/p65 pathway in Lenti-shSOCS3-injected mice.

SOCS3 is distributed extensively in the CNS including neurons, oligodendrocytes, astrocytes, and microglia. In addition, the SOCS3/Stat3 pathway has been implicated in regulating axonal regeneration, remyelination, and neuronal survival (31, 32). In our study, however, silencing of *SOCS3* occurred primarily in activated microglia and infiltrating macrophages. Therefore, we believe that the improvement of functional recovery was due to the ameliorated inflammatory microenvironment induced by the anti-inflammatory effects of silencing *SOCS3* in microglia/macrophages. It has been documented that M1 macrophages are toxic to neurons and that they can directly induce neuronal death, while M2 macrophages promote axonal growth (33, 34). Consistent with this, we previously observed that overexpression of BDNF at the lesion site of spinal cord injury increased the proportion of the M2 macrophages, which contributed to a notable improvement of functional recovery (35). In addition, Sarhane et al. reported that macroporous nanofiber wrap shifted the macrophage polarization toward a pro-regenerative M2 phenotype at the repair site and concomitantly improved axonal regeneration in a rat sciatic nerve cut model (36). Furthermore, several recent reports documented that drugs and transplantation of stem cells improved neurological functional recovery and prevented inflammation-induced demyelination by promoting microglial M2 polarization and inhibiting pro-inflammatory cytokine expression (37–40). Therefore, it is possible that driving differentiation of macrophages toward the anti-inflammatory M2 phenotype may provide a potentially therapeutic approach for the treatment of CNS inflammatory diseases. A limitation of the present study is that functional recovery and inflammatory responses were examined only at 14 days post-ICH, so further investigation is required to assess the long-term outcomes.

In conclusion, SOCS3 is a negative feedback molecule of Stat3-mediated signaling and plays critical roles in the regulation of the inflammatory response. Our observations suggest that SOCS3-silenced microglia/macrophages have a striking bias toward the M2 phenotype and have notable anti-inflammatory traits. The precise underlying mechanisms of these events remain to be fully elucidated. Furthermore, although lentiviral vectors have shown promising results in several clinical trials for treating X-linked adrenoleukodystrophy and leukemia, many challenges must be overcome for routine application of lentiviral vector-mediated gene treatment (41, 42). However, our results indicate that the infected cells were mainly microglia/macrophages around the hemorrhagic lesion area after the injection of the lentiviral vector into the hematoma cavity. In addition, as the inflammation subsided, lentivirus-infected macrophages gradually disappeared, suggesting that short-term viral gene therapy by injecting lentivirus vectors into the hematoma cavity may improve the safety of treatment.

## Data Availability Statement

All datasets presented in this study are included in the article/supplementary material.

## Ethics Statement

The animal study was reviewed and approved by the Fourth Military Medical University Committee for Animal Research.

## Author Contributions

GZ and M-ZC designed the study. The primary works come from X-CJ and Y-JS. The manuscript was written by X-CJ and revised by GZ. All authors contributed to the article and approved the submitted version.

## Conflict of Interest

The authors declare that the research was conducted in the absence of any commercial or financial relationships that could be construed as a potential conflict of interest.
